# Are tones in the expressive lexicon iconic? Evidence from three Chinese languages

**DOI:** 10.1371/journal.pone.0204270

**Published:** 2018-12-04

**Authors:** Arthur Lewis Thompson

**Affiliations:** Department of Linguistics, University of Hong Kong, Hong Kong, S.A.R., China; Leiden University, NETHERLANDS

## Abstract

Recent advances in the literature have focused on sketching phonosemantic mappings of imitative or iconic utterances by relying on vowels and consonants, leaving the suprasegmental information unexplored. To begin bridging this gap, this study looks at the interaction of lexical tone and iconicity by comparing sound symbolic (i.e., mimetic, expressive, ideophonic) strata and general (i.e., arbitrary, prosaic, non-iconic) strata from three Chinese languages (Mandarin, Taiwanese Southern Min, Hong Kong Cantonese) using corpus-based means. For all three languages the distribution of tones in the sound symbolic strata are skewed so that the majority of syllables are largely confined to two tonal categories per language, one of which is high level, while the general strata exhibit no such tonal bias. These results indicate that phonological systematicity at the prosodic level might play an important role in demarcating an iconic class of words. This cross-linguistic tendency towards high tone mappings may be derived from phonotactic strategies to facilitate prosodic foregrounding of iconic utterances as well as an embodiment of expressive voice and marked pitch use like that of Infant Directed Speech.

## Introduction

Certain linguistic structures involve imitative mechanisms. Onomatopoeia is one such example of iconicity or linguistic imitation, e.g. English *woof* (dog barking) or *bang* (explosion), which contradicts the long-held Saussurean principle of linguistic arbitrariness [1]. For example, the English *tree*, the French *arbre*, and the Polish *drzewo*, like many words, do not imitate their physical referent. However, a growing body of research shows that certain aspects of language, known collectively as sound symbolism, are formed through linguistic imitation of the outside world [2, 3, 4, 5, 6]. These findings have led to a deeper investigation into what exactly qualifies a word as iconic. A commonly held definition of iconicity is that the structure of a word is influenced by the structure of its referent, e.g., shape, sound, rhythm [7, 8, 9].

In this paper, iconicity is taken to refer to the (inherently) imitativeness or imitative properties of a phenomenon through perceptuo-motor analogy. In linguistic phenomena, iconicity can be found in certain types of gesture [10, 11], sign language, and spoken language. Here, of course, our concern lies with the iconicity of spoken language, i.e., spoken iconicity. Spoken iconicity can be further subdivided into two subtypes: unconventional and conventional iconicity [12]. Unconventional iconicity refers to iconic utterances that are essentially pure mimicry to the point of being non-linguistic [13, 14], whereby the orthography of a given language is hard-pressed to capture the phonetic characteristics of that utterance so that it might be understood, or repeated, by another speaker without explanation. Unconventional iconicity also has a tendency to violate what Attridge [15] calls ‘verbal structures’ or language-specific conventions for what constitutes a syllable, citing *pprrpffrrppffff* and *kraaaaaa* from James Joyce’s *Ulysses* as examples. More importantly, even though a speaker of English might be able to imagine potential referents of *pprrpffrrppffff* and *kraaaaaa*, there is no conventional, well-known, or immediate reference applicable to unconventional iconicity without an adequate supply of context. Conventional iconicity, on the other hand, mirrors unconventional iconicity. Firstly, conventional iconicity is linguistic and not purely mimicry. Although conventional iconicity is known to violate some phonotactic patterns in a given language [4, 12], (such as English *boing* where /ɔɪ/ as a diphthong does not otherwise occur before the velar nasal /ŋ/), it does not violate what constitutes the initial, nucleus, and coda positions of a syllable. For these reasons, conventional iconicity lends itself more easily to the orthography of a given language. Conventional iconicity also lends itself more easily to a referent without requiring the same amount of contextual information as that of unconventional iconicity. For example, whether read or heard in isolation, the referents of *bow-wow*, *boom*, *zoom*, *woof*, *meow*, and *bang* are by and large retrievable for English speakers.

Before proceeding any further, some clarification of terms is in order. Spoken iconicity of the conventional variety is commonly referred to in the literature as sound symbolism, ideophones, mimetics, and expressives [16]. The terms ‘sound symbolism’ and ‘iconic word’ is used hereafter to refer to spoken iconicity of the conventional variety, while iconic refers to the imitative properties and perceptuo-motor analogy inherent in sound symbolism.

The way in which phonological structure encodes iconicity is known as phonological iconicity, i.e., how linguistic sound is organized to imitate referent whilst remaining an acceptably formed word. Investigations into phonological iconicity focus on identifying phonosemantic mappings of linguistic segments onto real-world or iconic properties [2, 12, 17, 18, 19]. One such mapping, from Japanese, is that syllable-initial /p/ indicates tautness or abruptness, as in *putto* ‘a burst’, *pon* ‘striking a drum’, *pin* ‘sound of a string stretched taut’ [2]. Another well-attested mapping is high vowels encoding high-pitch or piercing referents [4, 12, 20,]. In this way, phonosemantic mappings mark a starting point for understanding how sound symbolism contributes to the structure of expressive speech and how language is used to mimic sensory perception.

So far, phonosemantic investigations into phonological iconicity are limited to the segmental level [6]. Little is known about how the prosodic level (e.g., lexical tone, intonation, or stress) contributes to phonological iconicity in natural language. Vowels and consonants co-occur with tone, so it is plausible that the pitch of a lexical tone (e.g., high, falling, rising) adds a further imitative dimension to iconicity. For example, falling tone could imitate a physical or emotional drop, just as the quick release of a bilabial stop like *p* might be equated to abruptness. Another possibility is that high tone correlates with referents of higher pitch or, given what we know from *kiki bouba* tests about high vowels patterning with small size [21], high tone might even exhibit a bias towards smallness.

It is also possible that lexical tone delineates one imitative property from another for a single segment. For example *p* associated to a high tone syllable could indicate abruptness, while *p* to a falling tone syllable could indicate another percept, such as tautness. This concept is similar to a phenomenon in Japanese mimetics whereby voiceless consonants can be systematically voiced to add intensity [2]: *korokoro* (rolling) > *gorogoro* (heavy or vigorous rolling). The overall meaning (rolling) remains the same, but the difference in voicing marks a contrast of perceptual intensity (not heavy vs. heavy). This concept is similar to minimal pairs in Mandarin where tone contrasts aspects of a shared general meaning [22]. Take the minimal pair [k^h^ân] *to look* and [k^h^án] *to keep watch* (both orthographically represented as 看) as an example. Here the segmental structure /k^h^an/ provides the semantic sense (vision) while the difference in lexical tone (high fall vs. high level) delineates two specific aspects of that sense. It is possible that tone in the sound symbolism of Chinese languages serves a similar purpose.

This paper addresses these gaps in our knowledge about lexical tone’s involvement with iconicity by reporting how lexical tone in three Chinese languages (Hong Kong Cantonese, Mandarin, and Taiwan Southern Min) is distributed within their respective sound symbolic inventories and lexicons at large. It is hoped that the findings reported in this study can serve as a baseline for future investigations into prosodic and tonal phenomena of sound symbolism.

Lexical tone is integral to phonological structure in Cantonese, Mandarin, and Taiwan Southern Min: every syllable bears one underlying lexical tone. (Even Mandarin unstressed clitics such as 了 l*e* PERFECTIVE and 的 *de* RELATIVE are specified for tone so that, if ever said in insolation, they are pronounced /liau213/ and /ti55/ respectively). However, due to the general lack of information on the relationship between lexical tone and iconicity, it is difficult to predict how tone might map onto iconicity for these three languages. What we do know is that prosody and sound symbolism interact. In their paper on expressiveness in Japanese ideophones, Akita and Dingemanse [23] show that Japanese sound symbolic expressions are often contrasted from the rest of the utterance with marked phonation, pitch, or intensity. While this contrastiveness is not phonological per se, prosodic markedness, or prosodic foregrounding, has also been noted in other languages and is generally assumed to convey an added sense of expressiveness to sound symbolic words [24–27]. It is unclear whether this added expressiveness or prosodic foregrounding is possible for the three tonal languages investigated here. As there has yet to be any formal investigation into the relationship of lexical tone and iconicity [3, 23], it remains unclear whether or how lexical tone is phonetically and phonotactically compatible with prosodic foregrounding. Before future investigations can answer this question, first, we must determine whether lexical tone contributes to iconic expression in these three languages.

The hypotheses here are that, for Mandarin, Hong Kong Cantonese, and Taiwan Southern Min, lexical tone (1) is either used to make phonosemantic mappings, like consonants and vowels at the segment level (e.g., /p/ = abruptness; falling tone = falling motion), (2) and/or it is used as a means for signalling iconic matter, like prosodic foregrounding. It should be noted that hypotheses (1) and (2) are not necessarily mutually exclusive.

It has been shown that the pitch-accent system of Japanese allows for prosodic foregrounding to take place in natural speech [23]. This is perhaps due to the binary nature of Japanese pitch-accent which is arguably not as restricted, in terms of contrasting contour or pitch level, as lexical tone is for Chinese. Moreover, pitch makes contrasts for grammatical function as well as aspectual eventualities in Japanese sound symbolism rather than iconic meaning [28].

Below are examples of sound symbolic words from each language which are minimally contrasted by tone. Tones are bolded and given in Chao tone letters (5 indicates the highest point of the pitch range and 1 indicates the lowest. 213 would thus indicate that the tone contour dips going from low (2) to lowest (1) and ending at mid (3). 55 and 11 indicates high level and low level tones respectively). Based on these examples alone, it is difficult to judge whether the tone assignment per language corresponds to hypotheses (1) and/or (2). See S1 Appendix for Chinese character list.

**Table:** 

**Mandarin** [29]	
p^h^ɤŋ **55** p^h^ɤŋ **55**	the sound of a heartbeat
p^h^ɤŋ **51** p^h^ɤŋ **51**	the sound of bumping
p^h^ɤŋ **35** p^h^ɤŋ **35**	the sound of a fierce wind
pɤŋ **55** pɤŋ **55**	the sound of palpitation, a bursting, or an explosion
pɤŋ **51** pɤŋ **51**	the sound or manner of jumping or hopping
**Hong Kong Cantonese** [30]	(underlined words are quotatives or arbitrary headwords)
tsi **55** tsi **55** sɛŋ 55	the sound of creaking (quotative: ‘emit sound’)
tsi **21** tsi **21** tsɐm **21**	the sound of whispering
la: **21** la: **35** sɛŋ 55	the manner of hurrying (quotative: ‘emit sound’)
ta:i 11 la: **21** la: **21**	the manner of being a great amount (headword: ‘large’)
ts^h^a:u 21 maŋ **33** maŋ **33**	the manner of being wrinkled or creased (headword: ‘crease’)
hak 55 maŋ **55** maŋ **55**	the manner of being pitch-black (headword: ‘black’)
**Taiwan Southern Min** [31]	(underlined words are quotatives, i.e., ‘to emit sound’)
hm **55** hm **55** kiɔ31	ground shaking during an earthquake
hm **33** hm **33** kiɔ31	thunder rumbling; roaring of wild animals
lin **31** loŋ **31** kiɔ31	sound of artillery
lin **51** loŋ **51** kiɔ31	sound or manner of rolling
lin **55** loŋ **55** kiɔ31	sound of jade or jewels clinking

The three languages tested here possess different lexical tone inventories respectively (Hong Kong Cantonese = 6 tones, Mandarin = 4 tones, Min = 7 tones) [32–34] as illustrated in Table 1.

**Table 1:** Descriptions of tonal inventory per variety of sinitic. Chao’s tone values given in parenthesis.

	T1	T2	T3	T4	T5	T6	T7	T8
Mandarin	High level (55)	Rising (35)	Dipping (213)	Falling (51)				
Hong Kong Cantonese	High level (55)	Mid rising (35)	Mid level (33)	Low falling (21)	Low rising (13)	Low level (11)		
Taiwan Southern Min (Taipei)	High level (55)	High falling (51)	Low falling (31)	Mid stopped (32)	Rising (14)	High falling (51)	Mid level (33)	High stopped (4)

To test our hypotheses, the distribution of lexical tones across an entire lexicon (referred to as general stratum of the lexicon) will be compared to the distribution within the sound symbolic stratum (all the sound symbolic words grouped as one distinct class). This leads to two possible outcomes. (1) If the distributions are comparable, then it is possible that lexical tone is an essential component of iconic expression (a detailed follow-up investigation would be required to see how the two converge, if at all). (2) If the distribution is not comparable, then it is still possible that lexical tone is indeed a component of iconicity but not an essential one. In this case, lexical tone may only be essential to iconic meaning in certain semantic realms, e.g., falling tone to depict falling actions. In the semantic realms where tone is not essential to iconic encoding, it is possible that tone is behaving according to some principle of systematicity [9], perhaps as a systematic marker or signal of iconicity. Likewise, it is equally possible that phonotactics, and not iconicity, would be the reason for certain tonal distributions as phonotactics are known to shape the syllabic structure of iconicity [3, 7]. What we found is that the tonal distribution across the three languages is skewed and consistent with our second prediction.

## Methods

This study is based on word lists (sound symbolic words) and corpus data (non-sound symbolic words). One list of sound symbolic words was consulted per variety of Chinese: Mandarin, Hong Kong Cantonese, Taiwan Southern Min. These word lists are taken to represent the sound symbolic stratum of each variety. Likewise, corpus data was consulted to represent the general (i.e., arbitrary, prosaic, non-sound symbolic) stratum per variety. In each corpus and word list, syllable types were counted per tone, separately for the sound symbolic stratum and the general stratum. For example, if we had a set of syllables like [ka4, ma1, ma3, ma3, mi1, mi1, mu2, pu1] (tones indicated by number) we would derive from this the following list of syllable types [mi1, ma1, ma3, mu2, ka4, pu1]. From the list of syllable types, we can see that tone 1 is the most common, i.e., 3 out of 6 syllable types are tone 1. From this we can deduce that the example set is skewed to tone 1. Once again, the goal of counting tones according to syllable types was to examine whether the tonal distribution is different between the sound symbolic stratum and the general stratum per Chinese language. A chi-squared test was used to compare tonal distribution between sound symbolic and general lexicon datasets. Statistical inference was corrected for multiple comparisons using Bonferroni correction across 20 tests (1 test of general distribution per variety, as well as 4, 6, and 7 single-tone tests for Mandarin, Hong Kong Cantonese, and Taiwan Southern Min respectively).

### Datasets

For each language, a sound symbolic word list was consulted to serve as an illustration of the sound symbolic lexical stratum of that language. Likewise, a corpus of spoken language was consulted to serve as an illustration of the general (i.e., prosaic, arbitrary, non-sound symbolic) stratum of the lexicon per language.

Sound symbolic words are colloquial in nature. They are known to occur in spoken narrative and story-telling [4, 5]. Therefore, we should compare sound symbolic words with non-sound symbolic words which also occur in colloquial settings (part of day to day speech). To create a snapshot of the tonal landscape for the general lexicon, we want to know which tones and how many of each tone a speaker might “encounter” in a colloquial setting (assuming this setting is composed of primarily non-sound symbolic words). Syllable type counts from spoken corpora were used to create this non-sound symbolic tonal snapshot. The goal here is to see how the proportion of tone types (i.e., how many falling tones?) in the general lexicon (colloquial, spoken corporal) stacks up against the sound symbolic stratum (word lists). Syllable types are counted according to their tonal assignment, e.g., 100 syllable types in high falling tone, 34 syllable types in rising tone etc.

An alternative method for illustrating the tonal snapshot for non-sound symbolic words would be to compare sound symbolic word lists with non-sound symbolic word lists. Non-sound symbolic word lists could be organized according to certain parameters, like grammatical category. In theory, this method seems most logical. However, such a method is problematic for the general lexicon. First of all, the goal here is to illustrate the tonal landscape of commonly spoken words rather than those commonly printed or those used in formal written style. This is perhaps less problematic for Mandarin given that it is the written standard in Mainland China. But written Cantonese relies heavily on stylized, so-called literary forms, as does Southern Min. Secondly, there is no clear line by which to delineate Chinese lexemes according to grammatical categories since many lexemes span multiple categories (i.e., noun, verb, adjective, adverb). For these reasons, this study instead uses syllable type counts taken from one spoken corpora of spontaneous speech per language.

All counts per dataset were done according to syllable type frequency. It follows that not all possible tone + syllable combinations are in use and some are probably not readily recognizable by a wide range of speakers (cf. §Discussion for tone gaps). The Mandarin sound symbolic stratum tone counts were compiled from the *Xiangshengci Cidian* (Dictionary of Onomatopoeia) [29], amounting to 188 different syllable types. The Mandarin general stratum tone counts were collected from a corpus-based survey of the 500 most frequent words, amounting to 759 different syllable types [35]. The Hong Kong Cantonese sound symbolic stratum tone counts were compiled from *A Corpus of Cantonese Ideophones*, amounting to 174 different syllable types [30]. Prior to counting, 73 syllables were excluded from the Cantonese corpus because they are in fact reduplicated adjectives and not sound symbolic words (e.g., reduplicated 怪怪地 gwaai3 gwaai3 dei2 ‘strangely’ from 奇怪 kei4 gwaai3 ‘strange’). The Hong Kong Cantonese general stratum tone counts were collected from the Hong Kong Cantonese Adult Language Corpus (HKCAC) [36], consisting of 1,923 syllables. HKCAC is a corpus of spontaneous Cantonese speech recorded during phone-in radio interviews and forums on Hong Kong radio. The Taiwan Southern Min sound symbolic stratum tone counts were compiled from a comprehensive survey of sound symbolic words from multiple Taiwanese dictionaries [31], amounting to 824 different syllable types. Taiwan Southern Min general stratum tone counts were collected from the Taiwan Southern Min (TSM) Corpus 1.0, an open access source, compiled by Ching Chu Sun and John Newman at the University of Alberta, which consists of 5,893 syllables comprising 747 different syllable types. This corpus is comprised of casual interviews with researchers about daily life topics. Only syllables from the interviewees’ speech (i.e., TSM Corpus A: Speakers 1, 2, 3, 4, 5, 6, 7.) were included in the data collection. It should be noted that Taiwan Southern Min syllables undergo a complex series of tone changes (i.e., tone sandhi) depending on their position in a multisyllabic word or phrase with relation to a head. The Taiwan Southern Min tones counted here are considered the *base* or *citation tones*—that is, the tone assigned to the syllable prior to the tone change, if any should apply.

## Results

**Fig 1:**
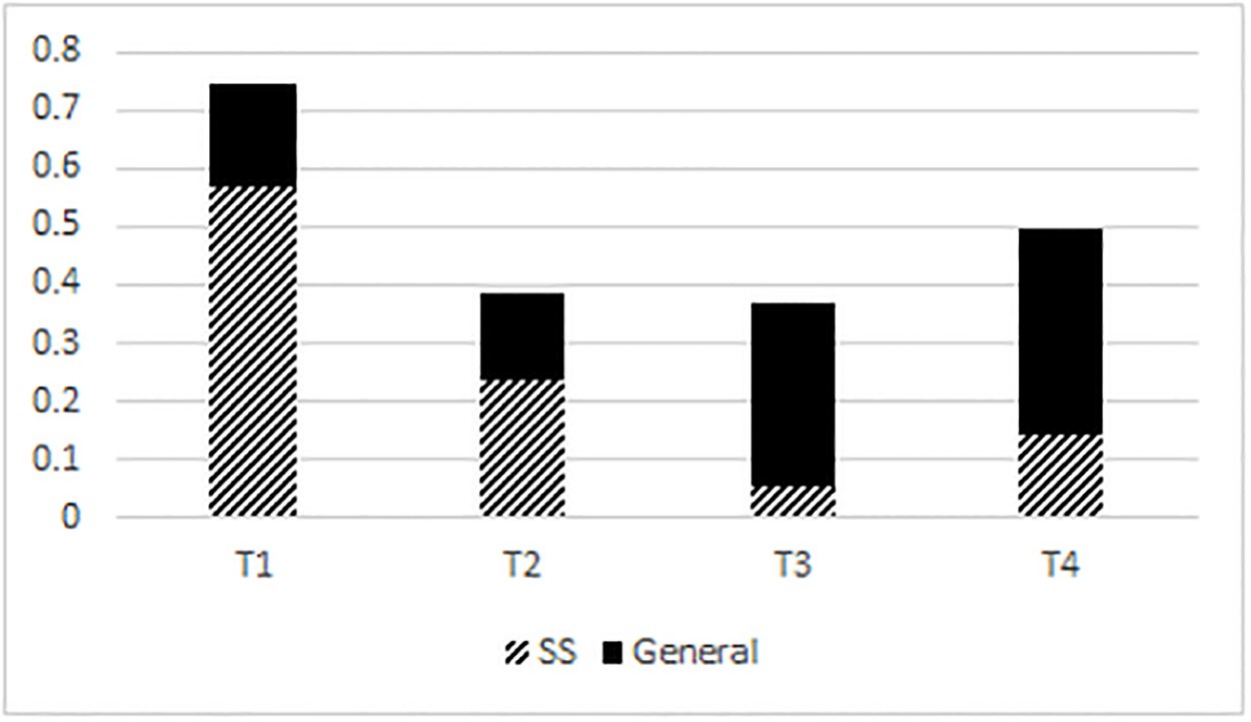
Tonal distribution in Mandarin. SS = sound symbolic, GL = general lexicon.

Fig 1 shows that the Mandarin high level tone (T1) is markedly prevalent within the sound symbolic stratum of the lexicon compared to all other tones (T2-T4). Fig 1 also shows that the distribution of tones within the general stratum of the lexicon is fairly balanced, with no tone making up the majority. Out of 188 sound symbolic tokens, 107 were in the high level tone (T1). The distribution of the high level tone between the sound symbolic stratum and the general stratum was significantly different (χ^2^ = 111.8, df = 1, p < .001). The distribution of all tones between the sound symbolic stratum and the general stratum was significantly different (χ^2^ = 147.4, df = 3, p < .001). In the sound symbolic stratum, T1 = 57% of all syllables, T2 = 23%, T3 = 5%, and T4 = 14%. In the general lexicon, T1 = 18% of all syllables, T2 = 15%, T3 = 32%, and T4 = 36%.

**Fig 2:**
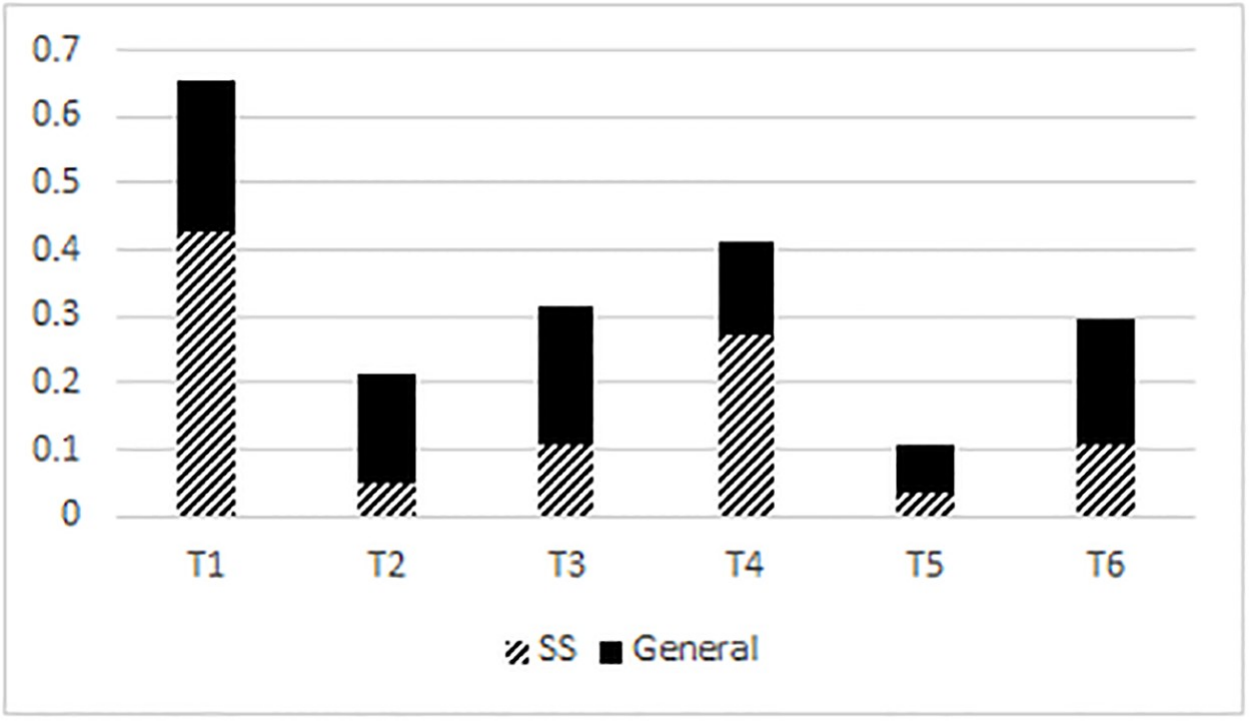
Tonal distribution in Hong Kong Cantonese. SS = sound symbolic, GL = general lexicon.

Fig 2 shows that the Hong Kong Cantonese high level tone (T1) and the low falling tone (T4) are markedly prevalent within the sound symbolic stratum of the lexicon compared to all other tones (T2, T3, T5, T6). It should be noted that the high level and low falling tones are historically derived from the same Middle Chinese *ping* tone category. Fig 2 also shows that the distribution of tones within the general stratum of the lexicon is fairly balanced, with no tone making up the majority. Out of 174 sound symbolic tokens, 74 were in the high level tone (T1), and 47 were in the low falling tone (T4). The distribution of all tones between the sound symbolic stratum and the general stratum was significantly different (χ^2^ = 71.43, df = 5, p < .001). The distribution of the high level tone between the sound symbolic stratum and the general stratum was significantly different (χ^2^ = 32.85, df = 1, p < .001). The distribution of the low falling tone between the sound symbolic stratum and the general stratum was significantly different (χ^2^ = 20.70, df = 1, p < .001). In the sound symbolic stratum, T1 = 43% of all syllables, T2 = 5%, T3 = 11%, T4 = 27%, T5 = 3% and T6 = 11%. In the general lexicon, T1 = 23% of all syllables, T2 = 16%, T3 = 21%, T4 = 14%, T5 = 7%, and T6 = 19%. It should be noted that Bodomo [30] did not specify entering tones (i.e., tones followed by a stop in coda position) T7, T8, and T9 in his corpus but instead listed them as their allophonic variants T1, T3, and T6 respectively.

**Fig 3:**
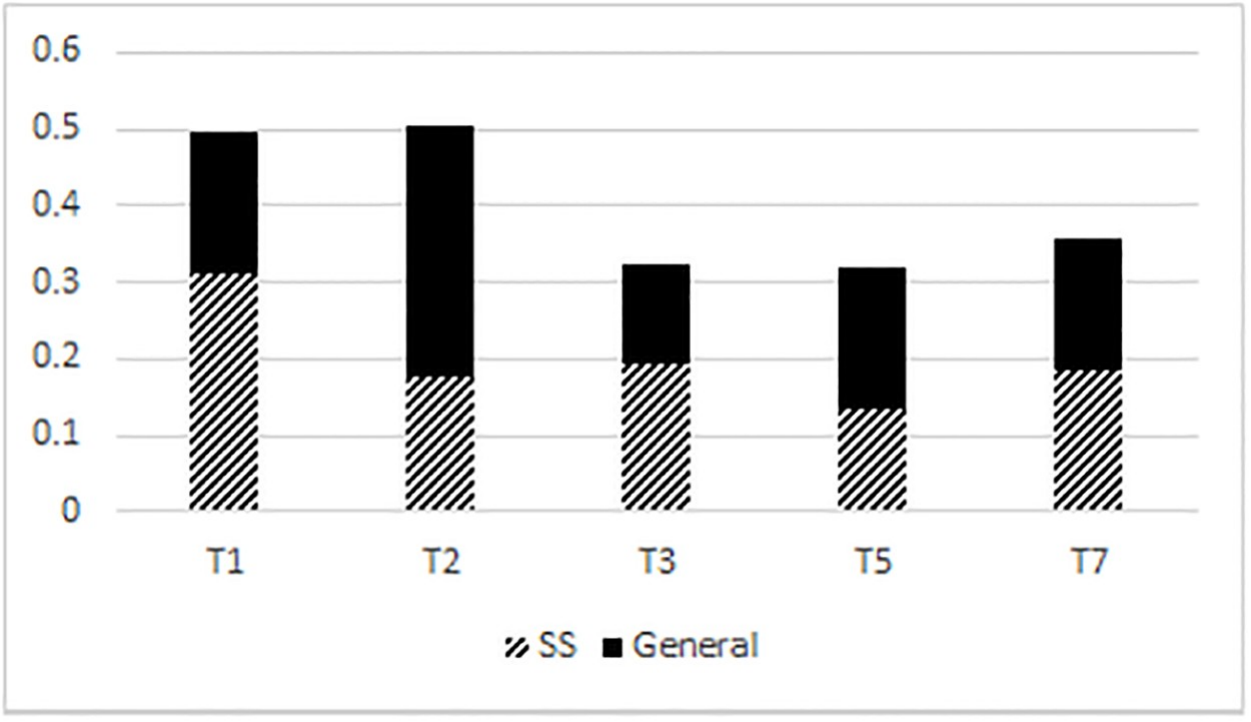
Tonal distribution in Taiwan Southern Min. SS = sound symbolic, GL = general lexicon.

Fig 3 shows that the Taiwan Southern Min high level (T1) and the low falling (T3) are markedly more prevalent in the sound symbolic stratum of the lexicon. However, the high falling tone (T2) is markedly less prevalent in the sound symbolic stratum of the lexicon compared to the general stratum of the lexicon. (The distributional results of the high falling tone (T2) here reflect the historical merger of T6 and T2 in Taipei Southern Min [34: 2691]. Because T6 and T2 are tonally the same, they were counted as one category). Out of 824 sound symbolic tokens, 164 were in high level tone (T1), 92 were in the high falling tone (T2), 102 were in the low falling tone (T3), 150 were mid level tones followed by a stop (T4), 70 were rising (T5), and 148 were high level tones followed by a stop (T8). The distribution of all tones between the sound symbolic stratum and the general stratum was significantly different (χ^2^ = 98.58, df = 4, p < .001). The distribution of the high falling tone (T2) between the sound symbolic stratum and the general stratum was significantly different (χ^2^ = 51.95, df = 1, p < .001). The distribution of the rising tone (T5) between the sound symbolic stratum and the general stratum was significantly different (χ^2^ = 9.64, df = 1, p = .021). In the sound symbolic stratum, T1 = 31% of all syllables, T2 = 17%, T3 = 19%, T5 = 13%, and T7 = 19%. In the general lexicon, T1 = 18% of all syllables, T2 = 33%, T3 = 13%, T5 = 19%, and T7 = 17%. The entering tones (i.e., tones followed by a stop in coda position) T4 and T8 were excluded from this analysis in Fig 3 as they take into account segmental information whereas the other tones do not. Unlike the Cantonese entering tones (so called T7, T8, T9), the Taiwan Southern Min tones cannot be collapsed into non-entering tone equivalents. This is because in Taiwan Southern Min, unlike Cantonese, entering tones are not allophones of non-entering tones.

**Fig 4:**
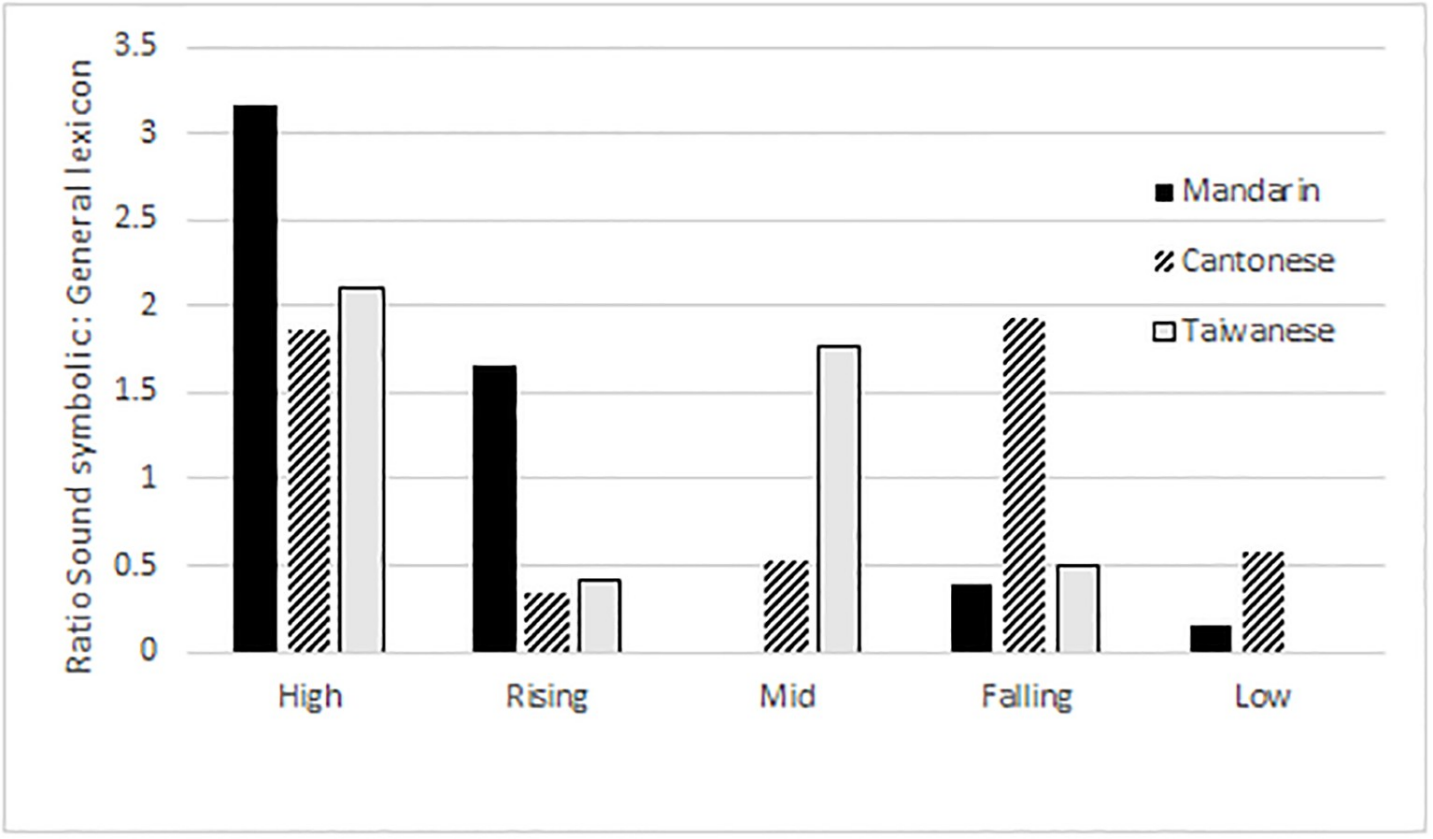
Relative percentage of tone across corpora.

Fig 4 shows the relative percentage of tone across the sound symbolic and general lexicon datasets from all three languages. For example, the proportion of Mandarin sound symbolic high tone to the general lexicon high tone is 3.16, meaning that high tone is 3.16 times more prevalent in the sound symbolic lexicon than in the general lexicon. For ease of cross-linguistic comparison, tones are compared according to general contour shape or pitch level. Language specific tones have been collapsed into broader categories, e.g., Cantonese low falling (T4: 21) and Taiwan Southern Min low falling (T3: 31) are both counted as ‘falling’ in Fig 4 despite their language specific differences. In Fig 4, Mandarin dipping tone (T3: 213) is considered low for phonological reasons [33]. Bars which surpass the number 1 labelled on the Y-axis indicate that a tone type is more prevalent in the sound symbolic stratum than the lexicon at large for that particular language. Fig 4 shows a general trend for sound symbolic words to have high tone. In fact, all three languages have exactly two tonal categories which are more prevalent in sound symbolic strata: Mandarin = high, rising; Cantonese = high, low falling; Taiwan Southern Min = high, low falling.

Figs 1–4 show that in Mandarin, Cantonese, and Taiwan Southern Min, the overall tonal distribution is significantly different between corpora, with the majority of all tone categories in the sound symbolic lexicons being skewed to a high level tone (Mandarin, Cantonese), a low falling tone (Cantonese), and high and low falling (Taiwan Southern Min). As Fig 4 shows, the relative percentage of tones across corpora is skewed to high as well as level tone categories.

## Discussion

Our predictions stated that (1) if the distribution of tones across the sound symbolic and general strata are comparable, then it is possible that lexical tone is an essential component of iconic expression; and (2) if the distribution is not comparable, then it is still possible that lexical tone is indeed a component of iconicity but not an essential one. In this case, lexical tone may only be essential to iconic meaning in certain semantic realms, e.g., falling tone to depict falling actions, or high tone to depict high pitch. In the semantic realms where tone is not essential to iconic encoding, it is possible that tone is a systematic signal of iconic words or that this tone is present for phonotactic reasons. Given that the results are in favour of prediction (2), I will now attempt to tease apart whether the skewed distribution of the results can be attributed to phonotactics (reduplication, tone gaps) or phonosemantics (iconic encoding). By phonosemantics, I mean that the assigned tone iconically encodes an imitative property of referent (e.g., high tone denotes a referent perceived as high pitch).

The results show that Hong Kong Cantonese sound symbolic words are skewed to high level tone (T1: 55) and low falling tone (T4: 21). From a phonosemantic standpoint, this is interesting because high level tone and low falling tone seem to make up opposite ends of the tonal spectrum and therefore might easily create depictive or iconic contrasts. What we cannot be sure of, without more detailed investigation into speakers’ perception of individual words, is whether these contrasts are actually considered depictive of the referent or not. For example, tone might be simply exploited as a means of homophone distinction (at the segment level) with little regard to depictive content. But given that there are words like tsi **55** tsi **55** ‘the sound of creaking’ and tsi **21** tsi **21** tsɐm **21** ‘the sound of whispering,’ where low falling tone is assigned to the ‘quieter’ referent (whispering), one could easily imagine that tone in Cantonese could be depictive in certain contexts.

From a phonotactic standpoint, one potential explanation for the marked appearance of the low falling tone is that reduplicative paradigms influence tone realizations. Even though low falling tone is still found in non-reduplicated disyllabic forms (e.g., examples (a) and (b) below), Tsou [37, 38] shows that low falling tone (21) syllables make up a majority of the paradigms for sound symbolic reduplication. This reduplication seems to be subject to a phonological constraint where high level tone cannot be realized as the rightmost reduplicated syllable if the following syllable is low falling tone. Since both (a) and (b) can be reduplicated as (c), (d), or (e), it seems that low falling tone becomes prevalent in the Cantonese sound symbolic inventory. More detailed investigation is needed to see whether the marked appearance of low falling (21) tone in Cantonese sound symbolism is due to iconic encoding (phonosemantics, i.e., low tone is perceived as imitative of the referent) or reduplicative paradigms (arbitrary phonotactics).

(a) piŋ 55 pa:ŋ 55 ‘two consecutive explosions’
(b) piŋ **21** pa:ŋ **21** ‘two consecutive explosions’

For both (a) and (b), the first syllable /piŋ/ is reduplicated as /piŋ.liŋ/ and the second syllable /pa:ŋ/ is reduplicated as /pa:ŋ.la:ŋ/ which can derive reduplicated forms according to different tone patterns shown in (c-e).

(c) piŋ 55 liŋ 55 pa:ŋ 55 la:ŋ 55 ‘many consecutive explosions’
(d) piŋ **21** liŋ **21** pa:ŋ **21** la:ŋ **21** ditto
(e) piŋ **21** liŋ 55 pa:ŋ**21** la:ŋ **21** ditto

Like Cantonese, Taiwan Southern Min sound symbolic words are skewed to high level tone (T1: 55) and low falling tone (T3: 31). While there seems to be no reduplicative explanation here for why high level and low falling are so prevalent, it should be noted that Taiwan Southern Min tones undergo a complex series of tone changes (i.e., tone sandhi) in multisyllabic contexts. Syllables to the left of the head of multisyllabic word or phrase will undergo a tone change. This would mean that the high level tone (T1: 55) changes to a mid level tone (33) and the low falling tone (T3: 31) changes to a high falling tone (51) when they are to the left a head syllable [39].

In Mandarin, the majority of sound symbolic words are in high level tone (T1: 55). As will be seen later in this section (cf. Table 2), this preference for high tone cannot be deduced to violations of historic or phonotactic tone gaps. From a phonotactic standpoint, there does not seem to be an obvious reason for high tone to be so prevalent in the sound symbolic inventory (e.g., Mandarin reduplication does not require reduplicated syllables or multisyllabic phrases to follow a paradigm which would account for this high level tone). Likewise, from a phonosemantic standpoint, it is difficult to discern what sort of referential property high tone might have. The following examples from the *Xiangshengci Cidian* [29], all of which are in high level tone, do not lead us to a phonosemantic mapping (e.g., high tone for referents which are perceived as ‘loud’ or ‘high pitch’ etc.). More perceptual input, in the form of native speaker judgements, is needed.

**Table 2:** Tone gaps in Mandarin, Cantonese, and Taiwan Southern Min.

	T1	T2	T3	T4	T5	T6	T7	T8	Total	Excluding Tones
Mandarin	333	257	319	349					1258	403 syllables
Gaps	70	146	84	54					354	
Cantonese	360	237	334	212	127	306			1143	587 syllables
Gaps	227	350	253	375	460	285			1205	
S. Min	421	400	362	276	389	T6 = T2	359	231	2207	883 syllables
Gaps	462	483	521	607	494	T6 = T2	524	652	3091	

**Table:** 

tɕi 55 tɕi 55 ʈʂa 55 ʈʂa 55	bird(s) chirping
tʊŋ 55 tʊŋ 55	beating a drum
tɤŋ 55 tɤŋ 55	sound of footfall; sound of a heavy object hitting the ground
ta 55 ti 55 ta 55 ti 55	sound of scraping
ta 55 ta 55	sound of gunfire
ts^h^ɹ̩ 55	sound of an object or person falling down
ts^h^a 55 ts^h^a 55	sound of footfall
pɤŋ 55	the sound of palpitation, a bursting, or an explosion

In this paper, tones were counted according to types instead of tokens. This was done because counting types can show us which tone + syllable types are possible and available. It follows that not all possible tone + syllable combinations are in use in common speech registers and are probably not readily recognizable by a wide range of speakers. Moreover, Mandarin, Cantonese, and Taiwan Southern Min syllable inventories all possess gaps where a tone + syllable should be segmentally possible but does not, in reality, occur. It is important that we check whether gaps like these account for the skewed distribution of tones shown in Figs 1–4. In this section, I will describe and enumerate the tone gaps (cf. Table 2) per Chinese language before showing how I account for them in my analysis of tonal distribution in sound symbolic strata.

Cantonese unaspirated onsets /p t ts k k^w^/ never occur with low rising (T5) or low falling (T4) tones, while aspirated onsets /p^h^ t^h^ ts^h^ k^h^ k^wh^/ never occur with the low level tone (T6) [40]. These two Cantonese tone gaps are systematic and without exception. Cantonese tone gaps were counted using the syllables listed in the *Zhonghua Xin Zidian* [41]. In Table 2, we can attribute the high number T1 syllables to the fact that T1, T3, T6 are the only tones that can also apply to syllables ending in plosives. T6 has the second highest number of syllables, followed by T3.

Unlike Cantonese, Mandarin tone gaps have many exceptions and thus cannot be said to be phonotactic. For this reason, segment specific tone gaps are not widely mentioned in the literature. In his thorough account of Mandarin phonology, Duanmu [33] only enumerates the tone gaps (cf. Table 2), and does not mention any interaction between tones and the segment level as Kirby and Yu [40] have done for Cantonese. However, by counting the syllable types listed in the *Zhonghua Xin Zidian* [41], I noticed one fairly consistent tendency: syllables with unaspirated plosive onsets /p t k / and nasal codas /n ŋ/ in high rising tone (35) are not common, but do still occur, e.g., 甭 beng35 ‘no need’, 哏 gen35 ‘ridiculous’. Though rooted in diachronic change [42, 43], this phonotactic tendency makes up for only 18 gaps out of the 146 gaps associated with the Mandarin high rising tone (cf. Table 2). Apart from this, there seems to be no other widely generalizable phonotactic patterns responsible for the tone gaps in Mandarin. Most gaps are either historical (diachronic change) or sporadic, a.k.a. “accidental” [44], gaps fulfilling no overt phonotactic constraint.

Taiwan Southern Min tone gaps were counted using *Tongiong Taiwanese Dictionary* [45]. Taiwan Southern Min does not seem to possess any outright phonotactic tone gaps. This is perhaps due to the prevalence of tone sandhi which would interfere with any outright phonotactic restrictions between tone and the segment level. Tone sandhi requires syllables to undergo tone changes depending on their position within a multisyllabic word or phrase [39]. If there were strict tone gaps in Taiwan Southern Min, then we might expect this to tone sandhi to be less pervasive for certain syllables or exhibit more irregularities and exceptions in patterning. However, it should be noted that T4 and T8 possess a high number of gaps as these tone categories only apply to syllables which end with a plosive.

The number of tone gaps (Table 2) and their phonological environments, cannot explain the skewed distribution of tones as depicted in Figs 1–4 between the general and the sound symbolic strata of Mandarin, Cantonese, and Taiwan Southern Min respectively. What tone gaps *can* explain is which tone + syllable combinations are impossible. The sound symbolic strata of a language are in line with the tone gaps of that language. Sound symbolic strata do not possess any tone + syllable combinations which violate tone gaps. In other words, Cantonese has no sound symbolic words in low tone with aspirated stops in onset position. Likewise, Mandarin lacks sound symbolic words in high rising tone with unaspirated onsets and nasal codas. On the other hand, tone gaps cannot necessarily explain is which tones are more likely to appear in token counts. The likelihood of one tone appearing versus another is dependent on the nature or strata (sound symbolic/general) of the lexicon used (or counted) among other factors. Even if the Cantonese corpora cited in this study were to be recounted so that all syllables are equal and without tone gaps (namely, no low rise or low fall with unaspirated stops in onset position, and no low level tones with aspirated stops in onset position) then 9 onsets (out of 19) would have to be excluded from this recount. It is not feasible to explain tone distribution within a given lexicon by way of tone gaps.

Because the sound symbolic words do not violate tone gaps of each language, this would indicate that they are inherited from a previous (historic) stage in the lexicon because they have undergone the same sound changes as the surrounding lexicon has, thus preserving tone gaps. However, this observation cannot apply to sound symbolic words which violate the phonotactics of the canonical syllabary altogether, such as Cantonese /fiŋ 11/ ‘shaking’ or ‘loosely hanging’ [30], or Mandarin /p^h^ju 55/ ‘shooting’ [46], and have no orthographic form as a result. It would be difficult to trace these forms historically due to their orthographic ambiguity. In Cantonese, labial fricative /f/ cannot pair with high vowels; in Mandarin, bilabial stops cannot be palatalized with rounded vowels. It is unclear whether all non-canonical syllables in each language conform to the tonal gaps of Table 2. Because they lack orthographic forms or Chinese character equivalents, non-canonical syllables are absent from dictionaries and thus difficult to pin down if not already reported in the linguistic literature [47].

As one reviewer mentioned, certain syllable types may restrict which tone can become part of a sound symbolic word. The reviewer pointed out that the Mandarin syllable type /taŋ/ has a tonal gap whereby the dipping tone (T3) never occurs. While this is true, this does not explain why /taŋ/ sound symbolic words occur mostly in high tone (T1) as opposed to rising (T2) or falling (T4). Granted, there are a few syllable types with only one possible tone, e.g., /nɤŋ 35/ ‘able’ or /kei 213/ ‘give,’ but these are exceptional. According to Duanmu [33] only 35 syllable types exist which have just one tonal realization 59 syllable types have 2 tonal realizations. In his analysis of Mandarin tone types, Duanmu [33] concludes that “most syllables have four or three tones each, and a small number of syllables have two or one tone each.” The reviewer went on to say that if a sound symbolic word were to come about for syllable types like /nɤŋ/ or /kei/, then the speaker would have no choice but to use the only available attested tone (/nɤŋ 35/, /kei/ 213) or create a totally new syllable, e.g., /nɤŋ 55/ or /kei 55/. The reviewer seems to imply that for Mandarin speakers to create a new syllable (with no orthographic form, no Chinese character equivalent) is highly unlikely. However, as mentioned previously, such non-canonical syllable types indeed exist and are in keeping with the systematic tone patterns described in our results section. Mandarin has forms like /p^h^ju 55/ ‘shooting’, ‘/p^h^ja 55/ ‘slapping; wham,’ /by 55/ ‘beeping’, /twaŋ 55/ ‘springiness; bewildered,’ which not only lack Chinese characters, but also violate phonotactic constraints on syllable structure [33, 46]. Indeed, sound symbolic words are cross-linguistically known to deviate from overarching phonological rules of the non-sound symbolic lexicon [4, 12]. Without reverting to Pinyin orthography, Mandarin speakers would be hard-pressed to find a (homophonic) character with which to convey these non-canonical syllables in written language. Since these syllable types are unorthodox, we would not expect restrictions as to their tonal assignment. Any tone should be a good candidate for these syllables, yet high tone is consistently found. Taking Mandarin as our point of reference for this question, as we see in Table 2, the preference for high tone does not boil down to high tone being the most common tone type. High tone (T1) accounts for 333 syllable types, while dipping tone (T3) and falling tone (T4) account for 319 and 349 syllable types respectively.

The main finding of this paper is that the tonal distribution of the sound symbolic inventory is skewed to one or two tone categories per language investigated. But what about the exceptions? In Mandarin there is only a small number of sound symbolic words which take the dipping tone (213) also known as T3. In the *Xiangshengci Cidian* (Dictionary of onomatopoeia) [29] only 18 entries possess syllables with the dipping tone. One might suppose then that the dipping tone is highly iconic and necessary for encoding the depictive content of these 18 sound symbolic words. However, upon closer inspection of each entry, it becomes clear that most of these 18 sound symbolic words are likely historically fossilized forms. Gong [29] quotes example sentences from textual sources alongside each entry. Most sources for these 18 sound symbolic words are from pre-Qing Dynasty literature (see Table 3). That is to say, most of these T3 sound symbolic words are from a predecessor of modern Mandarin (or Modern Standard Chinese) that was not quite what Mandarin is today. It is not certain whether these words were ever pronounced with a dipping tone, let alone used in a colloquial setting. Moreover, unlike the majority of modern Mandarin sound symbolic words [46], many of these T3 entries are orthographically opaque, making it difficult to know how they would be interpreted by modern-day Mandarin speakers. For example, /ku 35 ku 213/ ‘the sound of a birdcall’ [29] is written with the character 角 meaning ‘corner’ or ‘horn’ which is normally pronounced as /tɕiau 213/ or /tɕwe 35/ in modern Mandarin (interestingly, the Cantonese reading of 角 ‘corner’ is /kok33/). Orthographically, modern Mandarin sound symbolic words are usually composed of a mouth radical (a semantic component indicating the oral or onomatopoeic nature of the character) on the left and a radical that corresponds to the whole character’s pronunciation on the right, e.g., 噗 /p^h^u 55/ ‘the sound of spitting out one’s drink in surprise’ the mouth radical 口 plus the nearly-homophonous character 菐 /p^h^u 35/ ‘thicket.’ Orthography aside, out of these 18 sound symbolic T3 words, three are quoted from post-Qing 20th century sources: 隱隱 /in213.in213/ ‘rolling thunder’ [29], 卜卜赤赤 /pu 213 pu 213. ʈʂʰɻ̩ 55 ʈʂʰɻ̩ 55/ ‘sound of artillery hitting soil or rock’ [29], and 咿咿宛宛 /i 55 i 55 wan 213 wan 213/ ‘interpreting a foreign language’ [29]. Further investigation is required to ascertain just how recognizable and depictive these three entries would be for modern-day Mandarin speakers. It is possible that the authors of these 20th century texts were emulating older literary styles, as Van Hoey [48] notes that historical forms of Chinese sound symbolic words are often considered literary rather than colloquial due to their association with classical Chinese. It is quite possible that some of the forms in Table 3 have lost their iconic properties and are now preserved as descriptive literary devices rather than depictive expressions.

**Table 3:** Mandarin sound symbolic words containing T3 (213) or dipping tone [29].

Orthography	Pronunciation	Meaning	Textual Source	Era of Text
咿咿宛宛	i 55 i 55 wan 213 wan 213	Interpreting a foreign language	《八哥博士的歡迎會》 *Ba ge bo shi de huan ying hui*	20th century
殷殷	in 213 in 213	vibration, marching of a crowd	《史記蘇秦列傳》 *Shi ji su qin lie zhuan*	Western Han Dynasty; BCE 206—CE 9
隱隱	in 213 in 213	rolling thunder	《過嶺者》 *Guo ling zhe*	20th century
𨏈𨏈	in 213 in 213	bursting, carts clattering by, rolling thunder	《廣雅疏證》 *Guangya Annotations and Proofs*	Qing Dynasty; CE 1636–1912
隱隱轟轟	in 213 in 213 xʊŋ 55 xʊŋ55	rolling thunder	《伍子胥變文》 *Wu zi xu bian wen*	Tang Dynasty; CE 618–907
疙蹅蹅	kɤ 55 ʈʂʰa 213 ʈʂʰa 213	knocking or colliding	《燕青博魚》 *Yan qing bo yu*	Yuan Dynasty; CE 1217–1368
榖榖	ku 213 ku 213	rodent squeaking	《支諾皋》 *Zhi nuo gao*	Tang Dynasty; CE 618–907
汩汩	ku 213 ku 213	flowing water	《黑夜》 *Hei ye*	20th century
汩活	ku 213 kuo* 55	flowing water	《長笛賦》 *Chang di fu*	Eastern Han Dynasty; CE 25–220
古剌剌	ku 213 la 55 la 55	rolling thunder	《西遊記》 *Journey to the West*	Ming Dynasty; CE 1368–1644
古魯魯	ku 213 lu 55 lu 55	bubbling liquid	《忠義士豫讓吞炭》 *Zhong yi shi yu rang tun tan*	Yuan Dynasty; CE 1217–1368
古都都	ku 213 tu 55 tu 55	sloshing or churning	《西廂記》 *Xi xiang Ji*	Yuan Dynasty; CE 1217–1368
角角	ku 35 ku* 213	birdcall	《此日足可惜贈張籍》 *Ci ri zu ke xi zeng zhang ji*	Tang Dynasty; CE 618–907
朗朗	laŋ 213 laŋ 213	reading aloud in unison; chime of morning bell at dawn	《奉使常山早次太原呈副使吳郎中》 *Feng shi chang shan zao ci tai yuan cheng fu shi wu lang zhong*	Tang Dynasty; CE 618–907
卜卜赤赤	pu 213 pu 213 ʈʂʰɻ̩ 55 ʈʂʰɻ̩ 55	artillery hitting soil or rocks	《地雷陣》 *Di lei zhen*	20th century
不朗朗	pu51 laŋ213 laŋ 213	beat of a hand drum	《魔合羅 》 *Mo he luo*	Yuan Dynasty; CE 1217–1368
咋咋	tsa 213 tsa 213	magpie call	《三奪槊》 *San duo shuo*	Yuan Dynasty; CE 1217–1368
作作索索	tswo 35* tswo 35* swo 213 swo213	rats gnawing	《口技》 *Kou ji*	Qing Dynasty; CE 1636–1912

*asterisks indicate syllables not normally associated with orthographic form shown

Bodomo’s [30] *Corpus of Cantonese Ideophones* does not contain any sound symbolic words of questionably historic origin. Each entry provides a sentence from a fairly recent Hong Kong tabloid or broadsheet (e.g. *Apple Daily*) containing the given ideophone. Most of these sentences are direct quotations from interviewees as opposed to literary devices, narrative content, or news jargon. This means that Cantonese exceptions (i.e., sound symbolic words of rising, mid, and low tone) deserve further investigation to see if iconic depiction, tone sandhi, and/or homophony-prevention (for segmentally identical forms) is at play here. Unfortunately, the list of Taiwan Southern Min sound symbolic words amassed in Hung [31] does not provide quotes alongside each entry in her list. Instead, Hung [31] provides the 15 dictionaries from which her list was compiled, the oldest being *Jianming Taiyu Zidian* [49] and the most recent being *Taiyu Shunjian Rumen Cidian* [50]. Without detailed investigation of each entry on her list, it is difficult to guess whether Hung [31] took Taiwan Southern Min sound symbolic words of historic origin into account or not.

While diachronically iconic forms like those of Table 3 present problems for researchers attempting to assess the synchronic sound symbolic inventory of a language, for historical linguists they pose exciting questions for comparative research. For example, how have synchronic sound symbolic words deviated from historic forms, if at all? Do synchronic forms correspond to diachronic forms? And do sound changes between historic and synchronic sound symbolic words also pattern with the historical sound changes that have occurred throughout the general lexicon? Furthermore, historical reconstructions of the words in Table 3 would provide insight into phonosemantic patterns for sound symbolic words of older forms of Chinese, such as Middle Chinese [51]. If diachronic phonosemantic patterns are comparable with synchronic phonosemantic patterns of unrelated sound symbolic words (e.g., Mandarin: 角角 ku 35 ku 213 ‘birdcall’ *diachronic* vs. 咕咕 ku 55 ku 55 ‘cooing dove’ *synchronic*), such a finding would provide good argument for the articulatory and perceptuomotor analogies proposed as the driving force behind iconicity [9].

## Conclusion

As the results show, the distribution of tones in the general (i.e., arbitrary, prosaic, non-iconic) strata differs significantly from the distribution of tones in the sound symbolic (i.e., iconic, expressive, imitative, mimetic) strata of Hong Kong Cantonese, Mandarin, and Taiwanese Southern Min respectively. This distributional difference alone is enough to propose that tone behaves differently, or serves a different purpose, in the general stratum of a lexicon than it does in its counterpart: the sound symbolic stratum. As to what that different purpose may be, l propose the following two hypotheses:

1. The markedly prevalent tone category is a systematic signal for iconic matter
2. The markedly prevalent tone category is somehow readily iconic or facilitates iconic expression

The purpose served by tone in the general strata of Cantonese, Mandarin, and Taiwanese Southern Min, of course, is to differentiate lexemes which would otherwise be homophonous. A classic example is the Mandarin syllable /ma/ which can have four distinct meanings depending solely on tone assignment: /ma 55/ ‘mother,’ /ma 35/ ‘hemp,’ /ma 213/ ‘horse,’ and /ma 51/ ‘scold.’ However, tone assignments in the general lexicon do not seem to be systematic. That is to say, there is no apparent semantic reason (though there may be historically phonological reasons) why ‘mother’ is pronounced with a high tone as opposed to a rising, dipping, or falling tone, other than to distinguish it from homophonous words.

For the sound symbolic strata of Cantonese, Mandarin, and Taiwanese Southern Min, the purpose of tone distribution is unclear for a number of reasons. Firstly, the sound symbolic stratum should be iconic and therefore each word within the stratum should be inherently imitative of its referent. From a phonosemantic perspective, this would mean that each unit at the segment level of a syllable should have some imitative purpose. Tone, being part of a syllable, albeit at the prosodic level as opposed to the segment level, might also be assigned for such an imitative purpose. There is, however, the matter of minimal pairs. Though far less prevalent than in the general lexicon, some sound symbolic minimal pairs are differentiated by their tone alone. But is this tone assignment erratic, like that of the general stratum, e.g., Mandarin /ma/? Or, are there imitative motivations at play? Given the limited number of such sound symbolic minimal pair examples, it is difficult to tell whether the difference in tone is iconic or just an arbitrary assignment which distinguishes two otherwise homophonous words. The results of this paper bring us one step closer to answering this question.

Not only do the results show that tonal distribution differs between general strata and sound symbolic strata of Cantonese, Mandarin, and Taiwan Southern Min, but they also show that the sound symbolic strata of these languages are skewed to at least one tone category per language. Mandarin is skewed to a high level tone, while Hong Kong Cantonese and Taiwanese Southern Min are both skewed to high level and low falling tone categories. The fact that sound symbolic strata are skewed to specific tonal categories (cf. Figs 1–3) leads me to propose two hypotheses: (1) the markedly prevalent tone category is a systematic signal for iconic matter; and (2) the markedly prevalent tone category is somehow readily iconic or facilitates iconic expression. It is also possible that (1) and (2) are not mutually exclusive. Though some preliminary analysis was carried out in the Discussion section of this paper, further and more detailed investigation is required.

One route for further investigation is eliciting native speaker judgements for novel or newly coined sound symbolic words. Examining novel sound symbolic words has been one method used to investigate iconic properties of Japanese iconicity [52, 53, 54]. By asking speakers to invent novel sound symbolic words or to assign a given meaning to newly-created sound symbolic words, it might be possible to test whether a specific tone category is a systematic signal for iconic matter, i.e., hypothesis (1). For example, if Mandarin speakers consistently create new sound symbolic words in high tone (55), despite semantic differences between stimuli, then this would support an argument for high tone to act as some kind of systematic marking or delineator of iconicity as a word class. Future studies might also want to take into account syllable types which have limited tonal realizations. For Mandarin, Duanmu [33] reports that 35 syllables have only one tonal assignment. How many of these 35 types are also found in the sound symbolic stratum? Future studies might also want to consider which syllable types are totally absent from the sound symbolic stratum.

Testing whether a specific tone category facilitates iconic expression, i.e., hypothesis (2), is somewhat less straightforward. Dingemanse and Akita [23] have shown that variation in pitch, what they call *prosodic foregrounding*, over sound symbolic words of an utterance is a discourse strategy used to enhance the performative and depictive aspects of sound symbolic words in fluid Japanese speech. It is possible that something similar happens in Chinese languages. Perhaps the tone categories which are markedly prevalent in the sound symbolic inventory somehow facilitate performative and depictive prosody in fluid speech. A large spoken corpus, like that used in Dingemanse and Akita [23], would be needed to test whether pitch over the sound symbolic words of Chinese languages does indeed behave in a manner comparable to Japanese prosodic foregrounding.

From a strictly phonosemantic perspective, based on the results of this paper, it seems that tone cannot be easily collapsed into a semantic category of iconic depiction. Tone in these Chinese languages does not seem to map as neatly as some classic cross-linguistic examples of phonosemantic mappings do, e.g., nasal finals for encoding reverberation, high vowels for encoding high pitched or piercing sounds, or syllable-final (stop) consonants for encoding abrupt terminations of sound [4, 12, 20]. In the languages examined here, most of the sound symbolic words, regardless of depictive meaning (path, pitch, animal call, emotional state, reverberation, abruptness etc.), were almost completely exclusive to one or two tone categories. While there are a few exceptions to the markedly prevalent tone per language (cf. minimal pairs listed in §Introduction; Mandarin dipping tone in §Discussion), it is difficult to tell whether the tone assignment on exceptional cases is arbitrary or iconic. More exceptions are needed to verify iconic mappings along with native speaker input, perhaps. For example, the Cantonese sound symbolic strata is skewed to both high and low falling tone. One reason for the marked prevalence of low falling tone could be arbitrary Cantonese reduplicative paradigms [37, 38]. However, since these tones are at opposite ends of the Cantonese tonal spectrum (high vs. low), it is tempting to imagine the iconic depictions two such opposing tonal categories could create. Detailed investigation comparing the semantic nuances of high vs. low falling tone should be undertaken and speaker judgements about the differences between tonally contrastive minimal pairs should also be collected in order to better understand the roles of these tones in Cantonese sound symbolism.

Due to the nature of sound symbolic data collection in this paper (i.e., word lists), we have also seen that some exceptions, like the dipping tone (213) found in 18 Mandarin sound symbolic words, could actually be the result of fossilized forms which present-day speakers might no longer consider iconic. The need for more evidence is somewhat of a paradox since iconicity implies that sound symbolic words should theoretically act as stand-alone icons. Nevertheless, several instances of similarly iconic forms (in this case, same lexical tone + similar meaning) are needed to verify whether or not a given property of a sound symbolic word is indeed iconic.

Chinese languages aside, the current study has set a baseline for future cross-linguistic investigations into the relationship of lexical tone and sound symbolism. The results of this paper might also reflect a linguistic tendency or preference for iconic words to co-occur with a prosodic high regardless of whether a language is tonal or not. Future studies looking into the iconicity of non-tonal languages should measure F0 phenomena, to see how F0 mappings compare to that of tone in lexical tone languages. A final note, with regards to language acquisition, given the high frequency of onomatopoeic utterances, as well as the exaggerated use of pitch in Infant Directed Speech [6, 55–58], future studies might also consider how the prosody of Infant Directed Speech might influence the production and acquisition of lexical tone in sound symbolic words.

## Supporting information

S1 Appendix List of Chinese characters. (PDF)

S1 File Containing raw data chi statistics for all figures. (XLSX)
