# Supplementary material for: Are tones in the expressive lexicon iconic? Evidence from three Chinese languages
Source: PLoS One. 2018 Dec 4;13(12):e0204270. doi: 10.1371/journal.pone.0204270 (PMC6279048; doi:10.1371/journal.pone.0204270)
Supplement: S1 Appendix — (PDF) [file pone.0204270.s001.pdf]

S1. Appendix Chinese Character List. White squares (i.e., □) represent syllables which have no standard written form or lack a corresponding character. All data points are available via corresponding language dictionaries and word lists cited in the references section.

Mandarin

怦怦	the sound of heartbeat
砰砰、碰碰	the sound of bumping
蓬蓬	the sound of fierce wind
嘣嘣	the sound of palpitation, a bursting, or explosion
蹦蹦	the sound or manner of jumping or hopping (e.g., boing)
嘰嘰喳喳	birds chirping
咚咚	beating a drum
噔噔	sound of footfall; sound of a heavy object hitting the ground
嘀嗒嘀嗒	sound of scraping
嗒嗒	sound of gunfire
刺	sound of an object or person falling down
嚓嚓	sound of footfall
嘣	the sound of palpitation

Hong Kong Cantonese

吱吱聲	the sound of creaking (quotative: ‘emit sound’)
吱吱噚	the sound of whispering
□ □聲	the manner of hurrying
大□ □	the manner of being a great amount (headword: ‘large’)
皺□ □	the manner of being wrinkled or creased (headword: ‘crease’)
黑蚊蚊	the manner of being pitch-black (headword: ‘black’)

Taiwanese Southern Min

噉噉叫	ground shaking during an earthquake
噤噤叫	thunder rumbling; roaring of wild animals
鈴㖹叫	sound of artillery
玲瓏叫	sound of jade or jewels clinking
